# Ferrocene anchored activated carbon as a versatile catalyst for the synthesis of 1,5-benzodiazepines *via* one-pot environmentally benign conditions

**DOI:** 10.1039/d2ra00202g

**Published:** 2022-05-17

**Authors:** Suman Kusuma, Komal N. Patil, Puneethkumar M. Srinivasappa, Nitin Chaudhari, Ajay Soni, Walid Nabgan, Arvind H. Jadhav

**Affiliations:** Centre for Nano and Material Sciences, Jain University, Jain Global Campus Bangalore 562112 India j.arvind@jainuniversity.ac.in jadhav.ah@gmail.com; Aragen Life Science Pvt. Ltd. (GVK Bioscience Pvt. Ltd.) Plot No. 284-A(Part) Bengaluru-562106 India; Department of Chemistry, School of Technology, Pandit Deendayal Energy University Gandhinagar Gujarat 382007 India; School of Chemical and Energy Engineering, Universiti Teknologi Malaysia Johor 81310 Malaysia; Departament d'Enginyeria Quimica, Universitat Rovira i Virgili Av Paisos Catalans 26 43007 Tarragona Spain

## Abstract

1,5-Benzodiazepine is considered as one of the central moieties in the core unit of most drug molecules. Construction of such moieties with a new C–N bond under solvent-free and mild reaction conditions is challenging. Herein, we present a benign protocol for one pot synthesis of 1,5-benzodiazepine derivatives by using ferrocene (FC) supported activated carbon (AC) as a heterogeneous catalyst. The catalyst FC/AC was characterized by several analytical and spectroscopic techniques to reveal its physicochemical properties and for structural confirmation. The synthesized catalyst FC/AC was explored for its catalytic activity in the synthesis of 1,5-benzodiazepines through condensation of *o*-phenylenediamine (OPDA) and ketones (aromatic and aliphatic) under solvent-free conditions. The robust 10 wt% FC/AC catalyst demonstrated appreciable activity with 99% conversion of diamines and 91% selectivity towards the synthesis of the desired benzodiazepine derivatives under solvent-free conditions at 90 °C in 8 h. Additionally, several reaction parameters such as catalyst loading, reaction temperature, effect of reaction time and effect of different solvents on selectivity were also studied and discussed in-depth. To understand the scope of the reaction, several symmetrical and unsymmetrical ketones along with different substituted diamines were tested with the synthesized catalyst. All prepared reaction products were obtained in good to efficient yields and were isolated and identified as 1,5-benzodiazepines and no side products were observed. The obtained catalyst characterization data and the activity studies suggested that, the synergetic effect occurred due to the uniform dispersion of ferrocene over the AC surface with numerous acidic sites which triggered the reaction of diamine and ketone to form the corresponding benzodiazepine derivative and the same was illustrated in the plausible mechanism. Furthermore, the synthesized catalyst was tested for leaching and recyclability, and the results confirmed that catalyst can be used for up to six consecutive cycles without much loss in the catalytic activity and its morphology which makes the process sustainable and economical for scale-up production. The present method offered several advantages such as an ecofriendly method, excellent yields, sustainable catalytic transformation, easy work-up and isolation of products, and quick recovery of catalyst.

## Abstract 2

1,5-Benzodiazepine is considered as one of the central moieties in the core unit of most drug molecules.

## Graphical abstract

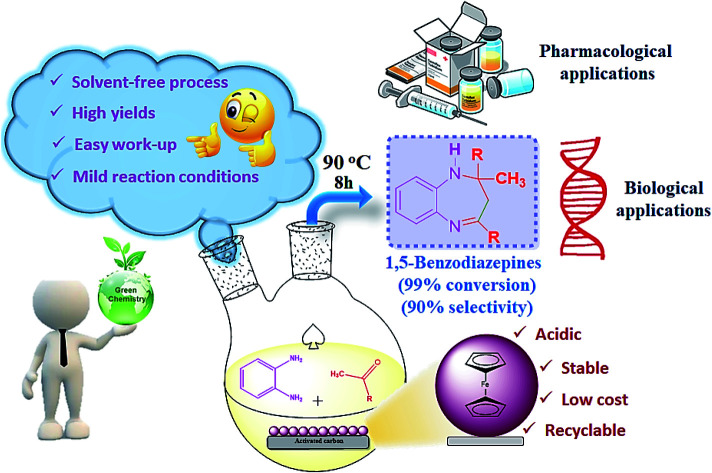

## 1. Introduction

Heterocyclic compounds occupy a prominent place in numerous pharmaceutically active intermediates.^1–4^ Amongst several compounds, benzodiazepine with nitrogen containing^6,7^ fused heterocycles is a well-established pharmacophoric scaffold.^5–10^ 1,5-Benzodiazepine and its derivatives form an important class of heterocyclic compounds exhibiting pharmacological and biological properties.^5–10^ Precisely, these derivatives have been known for their anti-convulsant, anti-anxiety, sedative, anti-depressive, anti-inflammatory, anti-microbial and hypnotic properties.^5–10^

In the last decade, the biological and pharmaceutical importance of 1,5-benzodiazepines has received more consideration and has been comprehensively investigated for several diseases such as cancer, cardiovascular disorders and viral infections.^5–10^ Additionally, 1,5-benzodiazepines have been considered as key intermediates in the synthesis of triazolo-, oxadiazolo-, oxazino- and furanobenzodiazepine type fused ring systems.^11^ Besides this, benzodiazepine derivatives can also be used as dyes for acrylic fibres in photography which demonstrates their significant commercial importance.^11^

Discovered in the 1970s, the benzodiazepine and its derivatives have since been studied extensively owing to their versatile applications.^9,10,12^ Numerous reviews are available in literature that represents different synthetic protocol for synthesis of 1,5-benzodiazepines.^5–7,9,10,13^ Researchers have reported synthesis of 1,5-benzodiazepines *via* domino reaction of *o*-PDA with α,β-unsaturated carbonyl compounds, or ketones.^12^ Additionally, enamino-benzodiazepines synthesis was also reported from 1,3,5-triketones or from allene-1,3-dicarboxylates and *o*-phenylene diamines (*o*-PDAs).^14^ Recently, one pot synthesis of 1,5-benzodiazepines *via* three-component reactions has also been extensively studied.^15^ Researched have also explored heterogeneous catalysts for synthesis of 1,5-benzodiazepines derivatives from diamines and acetone.^16,17^ Interestingly, benzodiazepines synthesis has also been reported by condensation of *o*-phenylenediamine (OPDA) with different carbonyl compounds in the presence of several acidic regents and catalysts which has attracted many researchers worldwide.^18,19^ It has been evidenced from the literature that presence of acid catalysts is very important to drive condensation process.^18,19^ Therefore, numerous acidic reagents such as boron trifluoride etherate, polyphosphoric acid, ytterbium(iii) triflate, gallium(iii) triflate, lead nitrate, acetic acid under microwave conditions and ionic liquids have also been used for the synthesis of benzodiazepines.^21–26^

However, almost all the reagents suffer major drawbacks such as high cost, high acidity and less eco-friendly. To over these drawbacks, in recent years researchers have started exploring activity of different solid acid catalysts such as sulfated zirconia, Al_2_O_3_/P_2_O_5_, Ag_3_PW_12_O_40_, PVP–FeCl_3_, and zeolite catalysts towards synthesis of benzodiazepines by condensation of *o*-phenylenediamine (OPDA) with different carbonyl compounds.^20,27^ However, even these catalysts suffered certain drawbacks such as requirement of harsh reaction operating conditions, catalyses side reactions, prolonged reaction time, low yields, and tedious workup procedure.^20,28,29^ Besides this, these reported solid acid catalysts demonstrated poor physicochemical properties such as low surface area, poor crystallinity, less porosity, more aggregated particles which make these catalytic materials unsuitable for their application in the synthesis of benzodiazepines.^28,29^

**Scheme 1:**
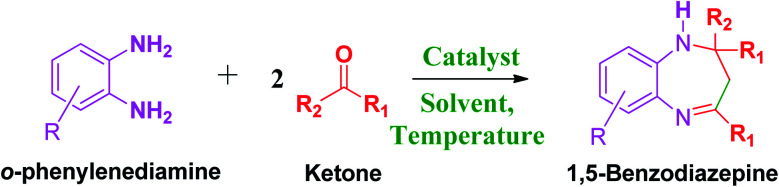
General reaction for synthesis of benzodiazepines by condensation of *o*-phenylenediamine with different carbonyl compounds.

These research gaps inspired the researchers world-wide to develop better catalysts with improved textural properties so as to achieve high activity towards the synthesis of 1,5-benzodiazepines under mild reaction conditions. In last couple of years, supported catalyst materials have received tremendous consideration as they offer several advantages over unsupported catalyst material (Scheme 1).^25^

In this context, ferrocene-based catalyst have fascinated scientists of various disciplines as ferrocene is one of the most stable, water insoluble, accessible, and low-cost organometallic compound with decent redox and catalytic properties.^28^ However, very few reports exist demonstrating the use of ferrocene-based catalysts for organic transformations.^30–34^ Pucheault *et al.* reported borylation of diazonium salts in the presence of catalytic amounts of ferrocene.^30^ Ferrocene was also used as a catalyst for decarboxylative cross coupling with toluene by Mao and group.^29^ There also exist reports on ferrocene catalyzed C–H imidation of arenes.^33^ Ferrocene-based ionic liquid supported catalysts have also been explored for the synthesis of naphthopyran derivatives.^34^ However, to the best of our knowledge, ferrocene/activated carbon has not been reported for its application in synthesis of benzodiazepine derivatives by condensation of OPDA with ketones (aromatic and aliphatic).

In the present work, we report facile synthesis of ferrocene supported activated carbon as sustainable catalyst for its applications in solvent free synthesis of 1,5-benzodiazepine derivatives under ecofriendly conditions. The synthesized catalyst FC/AC was characterized by advanced number of characterization techniques in order to gain insight in to its physicochemical properties and structural features. Further, the catalyst was investigated for its activity towards synthesis of 1,5-benzodiazepine derivatives by condensation of OPDA with ketones under solvent free condition. Interestingly, 10 wt% ferrocene/activated carbon demonstrated appreciable catalytic performance towards synthesis of 1,5-benzodiazepine with 99% conversion and 91% selectivity of desired product at 90 °C for 8 h under solvent free condition. Additionally efforts were paid to determine the effect of reaction parameters such as reaction temperature, catalyst loading, reaction period, solvent effect. On the other hand substrate scope was also determined by using substituted amines and both cyclic and acyclic ketones. All the reactions succeeded effectively for the production of 1,5-benzodiazepine derivatives in good yields. Further, the catalyst represented appreciable recyclability performance up to six recycles without significant loss in its catalytic activity and topography. Hence, the present approach offered use of highly active and stable ferrocene/activated carbon catalyst towards synthesis of 1,5-benzodiazepine derivatives without the use of hazardous organic solvent and in eco-friendly condition which would be an important step towards green and sustainable synthetic chemistry.

## 2. Materials and methods

### 2.1. Materials

Ferrocene (FC, purity 98%) was procured from Sigma Aldrich, India. Commercial activated carbon (AC, purity 98%) was also purchased from Sigma Aldrich, India. Acetonitrile (99%), methanol (99%), tetrahydrofuran (99%), *n*-hexane (99%), ethyl acetate (99%), pet ether (99%), *n*-pentane (99%) and chloroform solvents (purity 99%) were supplied by Sd Fine Chem. Limited. All substituted *o*-phenylenediamines and different substituted aliphatic and aromatic ketones were obtained from Sigma Aldrich, India (purity 99%).

### 2.2. Synthesis of ferrocene supported on activated carbon catalyst

#### 2.2.1. Synthesis of ferrocene supported activated carbon (FC/AC)

Ferrocene supported activated carbon was synthesized by using the given procedure below. Precisely, an appropriate amount of activated carbon was dispersed in 20 mL acetonitrile solvent by ultra-sonication for an hour. Subsequently, the required amount of ferrocene was mixed in the dispersed solution of activated carbon and stirred for about 15–30 min at rotatory evaporator. The excess solvent in the mixture was then evaporated by using rotatory evaporator (Buchi R-100) and the residual solid material was dried at 80 °C for 6 h to obtain final supported catalyst FC/AC. A series of catalysts were prepared by varying ferrocene content from 5 to 20 wt% in the final catalysts. The catalyst was henceforth denoted as FC/AC. Based on the reaction results for further characterization 10 wt% of FC/AC supported activated carbon catalyst was used.

### 2.3. Characterization techniques

The physicochemical features of the synthesized catalyst were studied by using several advanced characterization techniques. The presence of functional groups in the prepared samples was analyzed by using FT-IR spectroscopy. PerkinElmer FT-IR spectrophotometer (Spectrum Two) instrument was used to record FT-IR spectra by using KBr (IR grade) pallet method. The information on crystalline nature and phase development were obtained from their respective powder X-ray diffraction (XRD) patterns recorded on X-ray diffractometer (XRD; Rigaku Japan) with Cu Kα radiation source (*λ* = 1.5406 Å). The spectra were recorded between the 10–80° of 2*θ* range and a scan rate of 2° min^−1^. The TGA analysis of pure ferrocene and FC/AC catalyst analyzed to understand their decomposition process. The thermal analysis was performed from room temperature to 800 °C at a heating rate of 10 °C min^−1^ under N_2_ flow on Scinco TGA N-100 instrument.

Field emission scanning electron microscopy (FE-SEM), was used to gain morphological and topographical information. Prior to the analysis, the samples were evenly coated on the carbon tape placed on aluminium metal stub. The sample on the stub was then sputtered by gold nanoparticles for 120 s and the analysis was executed by using field emission scanning electron microscope (FE-SEM) (JOELModel-JSM7). XPS analysis was employed to understand elemental composition as well as electronic state of the elements in the samples. X-ray photoelectron spectra (XPS) were performed on an X-ray photoelectron spectrometer (Perkin ElmerPHI1257) at 4 × 10^−10^ Torr pressure with Al Kα X-ray as the excitation source (1486.7 eV).

Brunauer–Emmett–Teller (BET) and Barrett–Joyner–Halenda (BJH) method was respectively used to evaluate the specific surface area and pore size distribution of the materials. The analysis was performed on Belsorp MAX instrument (BEL Japan) at the temperature of liquid nitrogen. The materials were degassed at 100 °C for 2 h in high vacuum before the analysis. Additionally, the acidic sites in the samples were quantified by using NH_3_-TPD analysis using pure ammonia gas (99.999%). An indigenous set-up which comprised of a quartz tube reactor with a six-port valve together with a thermal conductivity detector (TCD) (M/s. Mayura Analyticals Pvt. Ltd, India) was used with NH_3_ as a probe molecule.

^1^H-NMR spectra were recorded on Varian Gemini (400 MHz) spectrometer using CDCl_3_ as a solvent and tetramethylsilane (TMS) as an internal standard. ^13^C-NMR spectra were also recorded on 100 MHz in the same solvent. The reactions were monitored by thin layer chromatography (TLC) on silica gel plates using pet ether and ethyl acetate as a solvent system. The melting points of the respective samples were recorded using Sigma Scientific instrument.

### 2.4. General procedure for synthesis of 1,5 benzodiazepine derivatives over synthesized FC/AC catalyst

A mixture of OPDA (1 mmol) and ketone (2.2 mmol) was placed in a two necked round bottom flask and required amount of FC/AC catalyst was added to the reaction mixture at room temperature (R.T.). Later the reaction mixture was heated at 90 °C for 8 h using oil bath and the reaction progress was monitored by TLC. The reaction mixture was then cooled to room temperature and filtered through Whatmann filter paper to separate out the FC/AC catalyst. The reaction mixture was diluted with *n*-pentane to separate out the catalyst and the excess solvent was evaporated under reduced pressure to afford dry crude product. The obtained crude product was purified by silica gel (100–200) column chromatography using ethyl acetate/pet ether as an eluent in 95 : 5 ratio to obtain pure compound. The compounds were characterized by ^1^H NMR, ^13^C NMR and mass spectroscopy.

The filtered catalyst was overnight dried at 80 °C and was reused for next cycle. Additionally, after separation of the catalyst from the reaction mixture, leaching test was performed. Specifically, to this reaction mixture fresh reactants (OPDA (1 mmol) and ketone (2.2 mmol)) were added and reaction was performed under the optimized reaction conditions without adding catalyst. We did not observe any progress in the reaction suggesting that there is possibly no leaching of ferrocene from AC support in the reaction mixture that could trigger the conversion of added reactants.

#### 2.4.1. 2-Methyl-2,4-diphenyl-2,3-dihydro-1*H*-benzo[*b*][1,4]diazepine (1)

Yellow solid: mp 107–112 °C; yield: 81%.^8^

##### ^1^H NMR (400 MHz, CDCl_3_)

*δ* 7.61–7.57 (m, 4H), 7.33–7.24 (m, 2H), 7.23–7.17 (m, 3H), 7.08–7.03 (m, 2H), 6.85–6.83 (m, 1H), 3.52 (bs, 1H), 3.14 (d, *J* = 13.2 Hz, 1H), 2.97 (d, *J* = 13.2 Hz, 1H), 1.76 (s, 3H).

##### ^13^C NMR (100 MHz, CDCl_3_)

*δ* 167.9, 147.8, 140.3, 139.8, 138.3, 129.9, 128.8, 128.5, 128.2, 127.3, 126.5, 125.6, 121.9, 121.6, 73.9, 43.3, 30.1; LCMS: *m*/*z* 313.24 (M + H)^+^.

#### 2.4.2. 2,4-Bis(3-chlorophenyl)-2-methyl-2,3-dihydro-1*H*-benzo[*b*][1,4]diazepine (2)

Brown solid: mp 98–103 °C; yield: 72%.^35^

##### ^1^H NMR (400 MHz, CDCl_3_)

*δ* 7.59 (S, 1H), 7.51–7.46 (m, 2H), 7.39 (d, *J* = 8.0 Hz, 1H), 7.32–7.25 (m, 3H), 7.18–7.04 (m, 5H), 6.85 (d, *J* = 7.2 Hz, 1H), 3.45 (bs, 1H), 3.08 (d, *J* = 13.2 Hz, 1H), 2.90 (d, *J* = 13.2 Hz, 1H), 1.75 (s, 3H).

##### ^13^C NMR (100 MHz, CDCl_3_)

*δ* 166.2, 149.5, 141.3, 139.9, 137.7, 134.6, 134.5, 130.0, 129.8, 129.5, 128.9, 127.5, 127.4, 127, 126.3, 125.2, 124, 122.2, 121.7, 74.0, 43.2, 30.0; LCMS: *m*/*z* 381.21 (M + H)^+^.

#### 2.4.3. 2,4-Bis(4-fluorophenyl)-2-methyl-2,3-dihydro-1*H*-benzo[*b*][1,4]diazepine (3)

Yellow solid: mp 101–106 °C; yield: 88%.^35^

##### ^1^H NMR (400 MHz, CDCl_3_)

*δ* 7.59–7.50 (m, 4H), 7.30–7.25 (m, 1H), 7.08–7.05 (m, 2H), 6.90–6.83 (m, 5H), 3.40 (bs, 1H), 3.07 (d, *J* = 13.2 Hz, 1H), 2.88 (d, *J* = 13.2 Hz, 1H), 1.75 (s, 3H).

##### ^13^C NMR (100 MHz, CDCl_3_)

*δ* 166.5, 165.3, 163.2, 162.8, 160.8, 143.3, 140.3, 137.8, 135.7, 129.2, 128.6, 127.6, 127.5, 126.6, 122.2, 121.7, 115.3, 115.2, 115.0, 73.8, 43.3, 30.0; LCMS: *m*/*z* 349.37 (M + H)^+^.

#### 2.4.4. 2,2,4-Trimethyl-2,3-dihydro-1*H*-benzo[*b*][1,4]diazepine (4)

Yellow solid: mp 125–130 °C; yield: 90%.^3^

##### ^1^H NMR (400 MHz, CDCl_3_)

*δ* 7.14–7.11 (m, 1H), 6.99–6.97 (m, 2H), 6.74–6.72 (m, 1H), 2.98 (bs, 1H), 2.38 (s, 3H), 2.25 (s, 2H), 1.36 (s, 6H).

##### ^13^C NMR (100 MHz, CDCl_3_)

*δ* 172.3, 140.8, 137.9, 126.8, 125.5, 122.1, 121.7, 68.4, 45.1, 30.5, 29.9; LCMS: *m*/*z* 189.22 (M + H)^+^.

#### 2.4.5. 2,4-Diethyl-2-methyl-2,3-dihydro-1*H*-benzo[*b*][1,4]diazepine (5)

Yellow liquid; yield: 88%.^3^

##### ^1^H NMR (400 MHz, CDCl_3_)

*δ* 7.15–7.11 (m, 1H), 6.98–6.94 (m, 2H), 6.74–6.68 (m, 1H), 3.10 (bs, 1H), 2.62–2.57 (m, 2H), 2.21 (q, *J* = 12.8 Hz, 2H), 1.68–1.57 (m, 2H).1.26 (s, 6H), 0.96 (t, *J* = 7.6 Hz, 3H).

##### ^13^C NMR (100 MHz, CDCl_3_)

*δ* 175.8, 140.9, 137.9, 127, 125.3, 121.8, 121.7, 70.7, 42.1, 35.7, 35.6, 26.9, 10.6, 8.5; LCMS: *m*/*z* 217.27 (M + H)^+^.

#### 2.4.6. 2,3,9,10*a*-Tetrahydro-1*H*-spiro[benzo[*b*]cyclopenta[*e*][1,4]diazepine-10,1′-cyclopentane] (6)

Brown solid: mp 112–117 °C; yield: 65%.^3^

##### ^1^H NMR (400 MHz, CDCl_3_)

*δ* 7.32 (d, *J* = 8.0 Hz, 1H), 6.98 (t, *J* = 8.4 Hz, 1H), 6.78 (t, *J* = 6.8 Hz, 1H), 6.57 (d, *J* = 8.0 Hz, 1H), 3.98 (bs, 1H), 2.75 (t, *J* = 8.4 Hz, 1H), 2.63–2.59 (m, 2H), 2.04–1.46 (m, 12H).

##### ^13^C NMR (100 MHz, CDCl_3_)

*δ* 178.0, 139.0, 133.9, 132.1, 126.9, 119.2, 118.6, 67.3, 54.1, 39.3, 38.4, 33.3, 28.9, 24.2, 24, 23.4; LCMS: *m*/*z* 241.29 (M + H)^+^.

#### 2.4.7. 1′,2′,3′,4′,10′,11*a*′-Hexahydrospiro[cyclohexane-1,11′-dibenzo[*b*,*e*][1,4]diazepine] (7)

Brown solid: mp 118–122 °C; yield: 61%.^4^

##### ^1^H NMR (400 MHz, CDCl_3_)

*δ* 7.28 (s, 1H), 7.01–6.93 (m, 2H), 6.72–6.70 (m, 1H), 3.52 (bs, 1H), 2.58 (t, *J* = 6.8 Hz, 2H), 2.38–2.40 (m, 1H), 1.88–1.16 (m, 16H).

##### ^13^C NMR (100 MHz, CDCl_3_)

*δ* 176.5, 138.9, 138.5, 129.6, 126.2, 121.5, 121.4, 52.2, 40.9, 34.5, 33.2, 27.5, 27.0, 25.6, 24.5, 21.9; LCMS: *m*/*z* 269.69 (M + H)^+^.

#### 2.4.8. Mixture of 2,4-bis(3-chlorophenyl)-2,7-dimethyl-2,3-dihydro-1*H*-benzo[*b*][1,4]diazepine and 2,4-bis(3-chlorophenyl)-2,8-dimethyl-2,3-dihydro-1*H*-benzo[*b*][1,4]diazepine (8 & 8A)

Yellow solid: mp 96–101 °C; yield: 89%.^4^

##### ^1^H NMR (400 MHz, CDCl_3_)

*δ* 7.51–7.43 (m, 2H), 7.41–7.36 (m, 1H), 7.25–7.13 (m, 4H), 6.93–6.87 (m, 1H), 6.77–6.75 (m, 1H), (bs, 1H), 3.32 (bs, 1H), 3.10–2.85 (m, 4H), 2.34 (s, 6H), 1.73 (s, 3H), 1.72 (s, 3H).

##### ^13^C NMR (100 MHz, CDCl_3_)

*δ* 166.1, 164.9, 149.3, 141.3, 141, 140, 137.4, 136.8, 134.9, 134.4, 134.2, 131.7, 129.7, 129.6, 129.5, 129.2, 128.9, 128.7, 127.4, 127.2, 127.1, 127, 126.1, 125, 124.9, 123.8, 74, 73, 43, 42.9, 29.9, 29.5, 21, 20.5; LCMS: *m*/*z* 395.14 (M + H)^+^.

#### 2.4.9. Mixture of 2,4-bis(4-fluorophenyl)-2,7-dimethyl-2,3-dihydro-1*H*-benzo[*b*][1,4]diazepine and 2,4-bis(4-fluorophenyl)-2,8-dimethyl-2,3-dihydro-1*H*-benzo[*b*][1,4]diazepine (9 & 9A)

Yellow solid: mp 119–124 °C; yield: 90%.^4^

##### ^1^H NMR (400 MHz, CDCl_3_)

*δ* 7.60–7.49 (m, 8H), 7.2–7.19 (m, 1H), 7.13 (S, 1H), 6.93–6.85 (m, 10H), 6.75–6.73 (m, 1H), 6.64 (s, 1H), 3.40 (bs, 1H), 3.27 (bs, 1H), 3.10–3.02 (m, 2H), 2.90–2.85 (m, 2H), 2.34 (s, 6H), 1.74 (s, 3H), 1.73 (s, 3H).

##### ^13^C NMR (100 MHz, CDCl_3_)

*δ* 166.5, 165.4, 165.1, 165, 163, 162.6, 162.5, 160.5, 143.2, 140.4, 137.5, 137.3, 136.4, 135.8, 135.7, 135.5, 135, 131.6, 129, 128.9, 128.8, 128.6, 128.4, 127.3, 127, 122.6, 121.6, 121.5, 115, 114.9, 114.8, 114.7, 73.7, 72.8, 43.3, 43.1, 29.9, 29.5, 21, 20.5; LCMS: *m*/*z* 363.73 (M + H)^+^.

#### 2.4.10. Mixture of 2,2,4,7-tetramethyl-2,3-dihydro-1*H*-benzo[*b*][1,4]diazepine and 2,2,4,8-tetramethyl-2,3-dihydro-1*H*-benzo[*b*][1,4]diazepine (10 & 10A)

Brown liquid; yield: 84%.^36^

##### ^1^H NMR (400 MHz, CDCl_3_)

*δ* 7.03–6.53 (m, 6H), 2.89 (bs, 2H), 2.54 (s, 3H), 2.44 (s, 3H), 2.35 (s, 3H), 2.34 (s, 3H), 2.28 (s, 2H), 2.20 (s, 2H), 1.33 (s, 3H), 1.32 (s, 3H).

##### ^13^C NMR (100 MHz, CDCl_3_)

*δ* 172.7, 171.6, 141.1, 138, 137.9, 135.4, 135.3, 131.8, 127.1, 127, 126.2, 122.8, 122.1, 121.9, 68.5, 67.8, 45.3, 45.2, 30.6, 30.3, 29.9, 29.8, 21, 20.7; LCMS: *m*/*z* 203.24 (M + H)^+^.

#### 2.4.11. Mixture of 6-methyl-2,3,9,10*a*-tetrahydro-1*H*-spiro[benzo[*b*]cyclopenta[*e*][1,4]diazepine-10,1′-cyclopentane] and 7-methyl-2,3,9,10*a*-tetrahydro-1*H*-spiro[benzo[*b*]cyclopenta[*e*][1,4]diazepine-10,1′-cyclopentane] (11 & 11A)

Brown solid: mp 178–183 °C; yield: 85%.^8^

##### ^1^H NMR (400 MHz, CDCl_3_)

*δ* 7.24 (d, *J* = 13.6 Hz, 1H), 6.59 (d, *J* = 8.0 Hz, 1H), 6.38 (S, 1H), 3.94 (bs, 1H), 2.76 (t, *J* = 9.2 Hz, 1H), 2.60–2.57 (m, 2H), 2.25 (s, 3H), 2.12–2.09 (m, 2H), 1.96–1.59 (m, 10H).

##### ^13^C NMR (100 MHz, CDCl_3_)

*δ* 169, 134.8, 133, 128.5, 125.9, 125.6, 125.3, 122.5, 120.1, 115.9, 84.8, 67.6, 35.6, 34.7, 30.4, 25.8, 24.9, 20.8, 16.1; LCMS: *m*/*z* 255.39 (M + H)^+^.

#### 2.4.12. 7′-Methyl-1′,2′,3′,4′,10′,11*a*′-hexahydrospiro[cyclohexane-1,11′-dibenzo[*b*,*e*][1,4]diazepine] and 8′-methyl-1′,2′,3′,4′,10′,11*a*′-hexahydrospiro[cyclohexane-1,11′-dibenzo[*b*,*e*][1,4]diazepine] (12 & 12A)

Brown solid: mp 130–135 °C; yield: 90%.^8^

##### ^1^H NMR (400 MHz, CDCl_3_)

*δ* 7.16 (d, *J* = 8.0 Hz, 1H), 6.73 (d, *J* = 7.6 Hz, 1H), 6.50 (s, 1H), 3.8 (bs, 1H), 2.56 (t, *J* = 6.4 Hz, 2H), 2.40–2.37 (m, 1H), 2.28 (S, 3H), 1.89–1.19 (m, 18H).

##### ^13^C NMR (100 MHz, CDCl_3_)

*δ* 175.0, 138.5, 136.2, 135.9, 126.8, 122.1, 121.4, 52.9, 41.3, 34.5, 33.5, 27.9, 27.4, 25.6, 24.8, 21.9, 21.1; LCMS: *m*/*z* 283.72 (M + H)^+^.

#### 2.4.13. Mixture of 7-chloro-2,4-bis(4-fluorophenyl)-2-methyl-2,3-dihydro-1*H*-benzo[*b*][1,4]diazepine and 8-chloro-2,4-bis(4-fluorophenyl)-2-methyl-2,3-dihydro-1*H*-benzo[*b*][1,4]diazepine (13 & 13A)

Brown solid: mp 101–105 °C; yield: 92%.^8^

##### ^1^H NMR (400 MHz, CDCl_3_)

*δ* 7.57–7.48 (m, 4H), 7.29–7.25 (m, 1H), 7.05–6.98 (m, 1H), 6.93–6.84 (m, 4H), 6.83–6.75 (m, 1H), 3.38 (bs, 1H), 3.27 (bs, 1H), 3.13–3.06 (m, 1H), 2.91–2.85 (m, 1H), 1.76 (s, 3H).

##### ^13^C NMR (100 MHz, CDCl_3_)

*δ* 167.6, 166.5, 165.5, 163.3, 163, 160.9, 142.9, 141.4, 138.9, 138.4, 136.4, 135.5, 135.2, 131.3, 130, 129.4, 129.3, 129.2, 128, 127.5, 127.4, 127, 126.2, 122.6, 121.9, 120.8, 115.4, 115.3, 115.1, 73.8, 73.2, 43.5, 43.3, 30.3, 29.9; LCMS: *m*/*z* 383.3 (M + H)^+^.

#### 2.4.14. Mixture of 7-chloro-2,2,4-trimethyl-2,3-dihydro-1*H*-benzo[*b*][1,4]diazepine and 8-chloro-2,2,4-trimethyl-2,3-dihydro-1*H*-benzo[*b*][1,4]diazepine (14 & 14A)

Brown liquid; yield: 90%.^3^

##### ^1^H NMR (400 MHz, CDCl_3_)

*δ* 7.11 (d, *J* = 2.4 Hz, 1H), 7.04 (d, *J* = 8.4 Hz, 1H), 6.94–6.90 (m, 2H), 6.71–6.63 (m, 2H), 2.35 (s, 3H), 2.34 (s, 3H), 2.25 (s, 2H), 2.21 (s, 2H), 1.33 (s, 6H), 1.32 (s, 6H).

##### ^13^C NMR (100 MHz, CDCl_3_)

*δ* 173.9, 172.7, 141.7, 139.1, 138.7, 136.5, 130.2, 128.2, 126.9, 126.5, 125.2, 122.6, 121.7, 120.9, 68.5, 67.9, 45.2, 45.1, 30.6, 30.4, 29.9, 29.8; LCMS: *m*/*z* 223.18 (M + H)^+^.

## 3. Results and discusssion

### 3.1. FC/AC catalyst characterization

#### 3.1.1. FT-IR analysis

**Fig. 1:**
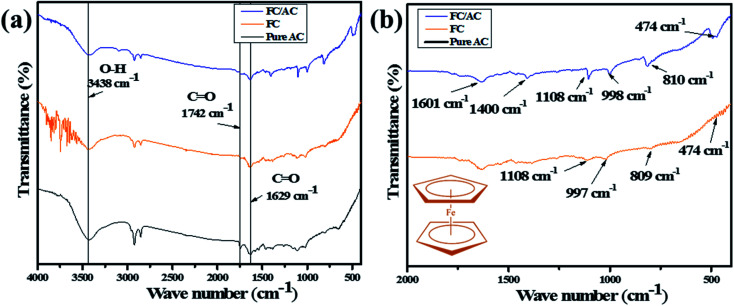
(a) FT-IR spectra of pure AC, FC and FC/AC and (b) fingerprint region of FC and FC/AC catalyst (inset of (b) ferrocene structure).

FT-IR spectroscopic technique was employed to confirm the presence of different functional groups in the synthesized materials.^37^Fig. 1(a) and (b) displayed the FT-IR spectra of pure activated carbon, ferrocene and FC/AC catalyst. The FT-IR spectrum of activated carbon (Fig. 1(a)) displayed broad band centered at 3438 cm^−1^ which can be attributed to O–H stretching vibration related to possible presence of hydroxyl, phenolic hydroxyl groups over the AC surface.^38^ The band at 1742 cm^−1^ can be assigned to C=O stretching vibration suggesting presence of carboxylic acid groups.^38^ Additionally, peaks observed at 1629 and 1400 cm^−1^ correspond to the stretching vibration of C=O bond deformation and C–O bond, respectively which further support the presence of carboxylic acid groups over the surface of AC.^38^ A weak peak at around 1108 cm^−1^ correspond to the stretching vibration of C–O indicating presence of alcohol, phenol ether or ester.^39^

The FT-IR spectrum of AC suggested presence of various oxygenated functional groups over its surface. Further, the peaks at 997 and 809 cm^−1^ appeared in the FT-IR spectrum of FC/AC relating to the out of-plane vibration of cyclopentadiene moiety of ferrocene.^40,41^ Additionally, a peak at 474 cm^−1^ corresponds to the stretching vibration due to the asymmetric ring metal.^40^ These peaks can be clearly observed in the finger print region (Fig. 1(b)) and are characteristic peaks corresponding to the presence of ferrocene. The peaks observed in FT-IR spectra of AC and FC were retained in the FT-IR spectrum of FC/AC suggesting successful formation of FC/AC catalyst.

#### 3.1.2. XRD analysis

**Fig. 2:**
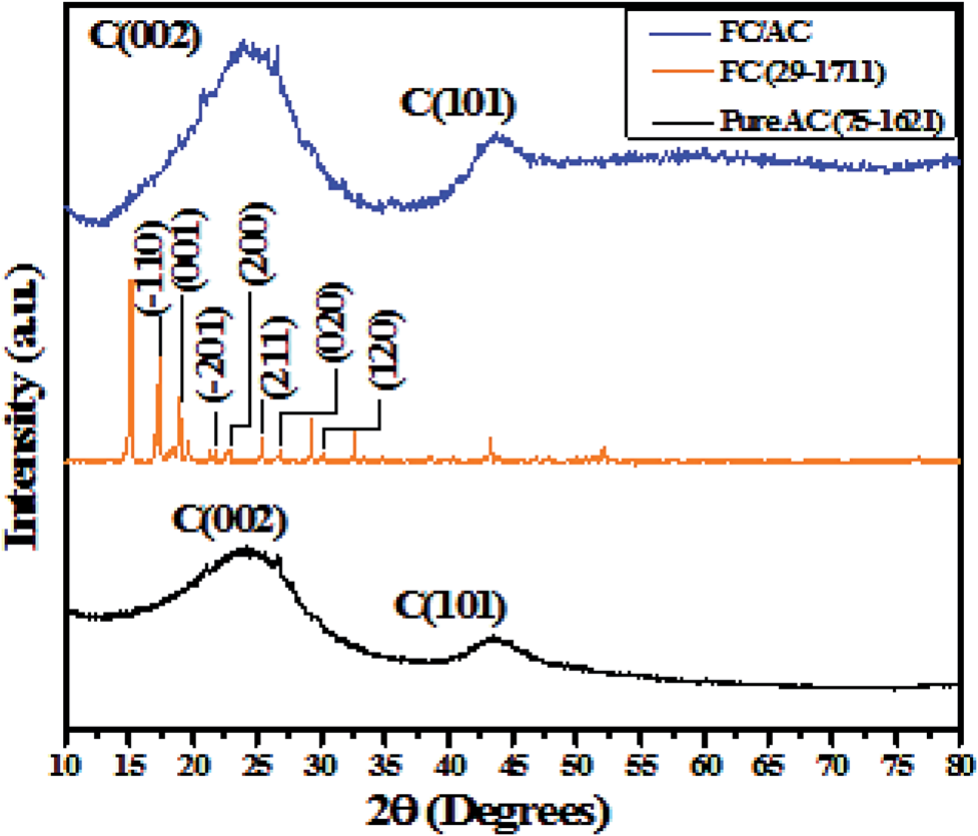
XRD pattern of pure AC, FC and FC/AC.

The XRD analysis of 10 wt% FeC/AC was performed along with pure AC and pure ferrocene to confirm the phase, crystalline nature and successful formation of the final FC/AC catalyst. The obtained XRD patterns of all the materials are displayed in Fig. 2. The XRD pattern of pure AC displayed two prominent diffraction peaks precisely at 25.8 and 43° corresponding to the (002) and (010) planes of graphitic carbon.^42,43^ The broad peaks indicated presence of amorphous carbon.^42,43^ Further, the XRD pattern of pure ferrocene displayed sharp diffraction peaks which indicated the crystalline nature of the ferrocene.^44^ Precisely, peaks were observed at 2*θ* values ∼17.5°, 19.1°, 28.9°, 22.9°, 25.3°, 26.8° and 30.1° which corresponds to the (−110), (001), (−201), (200), (211), (020) and (120), respectively.^44^ The XRD pattern of ferrocene well-matched with the JCPDS number 29-1711 which confirmed the monoclinic crystal structure of ferrocene.^44,45^

Additionally, XRD pattern of FC/AC is characterized by distinctive peaks derived from the AC and FC. However, low intense characteristic peaks corresponding to the ferrocene in the XRD pattern of FC/AC catalyst could be possibly due to low loading of ferrocene or presence of highly dispersed ferrocene molecules over the surface of AC.^46^ Meanwhile, it was observed that, intensity of peak corresponding to (002) plane of AC increased which suggested improvement in the crystallinity of activated carbon. This observation could possibly be due to the successful grafting of crystalline ferrocene on the activated carbon surface.

#### 3.1.3. TGA analysis

**Fig. 3:**
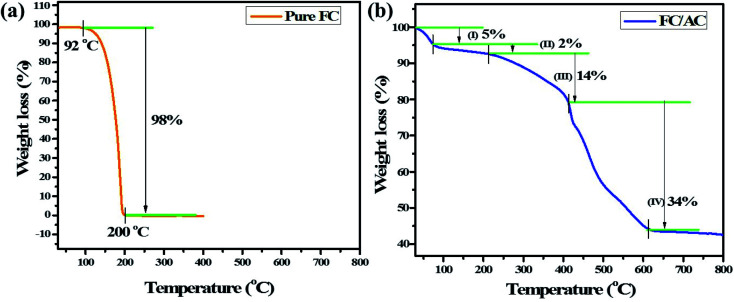
TGA analysis of (a) pure FC and (b) FC/AC catalyst.

The information on thermal stability of the FC/AC catalyst was obtained from TGA analysis. TGA analysis of pure ferrocene and FC/AC catalyst were performed and the obtained curves are shown in Fig. 3(a) and (b). Precisely, in the TGA curve of pure ferrocene (Fig. 3(a)) displayed only one major weight loss in the temperature range of 100–200 °C.^47–49^ Literature suggests that ferrocene experiences very slight sublimation at about 100 °C, and at around 400 °C further decomposes spontaneously into Fe, H_2_ and other hydrocarbons.^47–49^

On the other hand, TGA curve of FC/AC catalyst displayed four stage decomposition. To be specific, first 5% weight loss was observed up to 75 °C which could be attributed to the loss of adsorbed solvent molecules.^50^ The second weight loss (2%) occurred between 75 to 222 °C possibly due to loss of water of crystallization and water present in the lattice.^50–52^ A major weight loss between 222–430 °C in the FC/AC catalyst was ascribed to the decomposition of ferrocene along with the decomposition of surface functional groups of AC which was around 14%.^53^ Further, weight loss from 430 °C was due to initiation of decomposition of skeleton of activated carbon which contributed to 34% of total weight loss in the FC/AC catalyst.^26,29^ The description is well supported by literature reports.^50–53^

#### 3.1.4. FE-SEM and TEM analysis

**Fig. 4:**
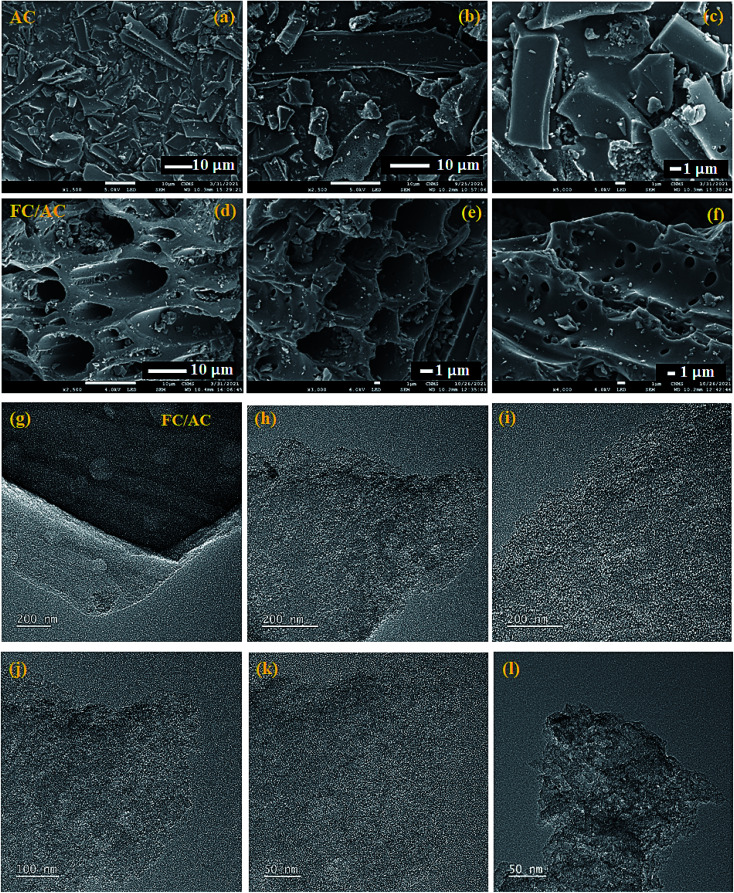
FE-SEM analysis of (a)–(c) pure AC and (d)–(f) FC/AC and TEM analysis of (g)–(l) FC/AC.

FE-SEM analysis was performed to obtain information on surface morphology of prepared catalyst.^37^ FE-SEM images of pure AC displayed in the Fig. 4(a)–(c) demonstrated thick structures with smooth with regular pore structure.^42^ Such a distinctive structure permits the activated carbon to have both high pore volume and large specific surface area. On the other hand, deposition of ferrocene on the AC resulted in coarse and irregular surface morphology (Fig. 4(d)–(f)). The regular pore structure observed in AC was destroyed. This may be due to ferrocene deposition in the pores of the activated carbon which resulted in the increase in the pore size.^54^

TEM analysis of FC/AC was performed to further confirm the morphology and the results are represented in Fig. 4(g)–(l). The TEM images of the FC/AC catalyst clearly demonstrated presence of ferrocene over activated carbon. The images were captured at different magnifications which clearly showed proper dispersion of ferrocene over support. The explanation is supported by the literature reports.^55–57^

**Fig. 5:**
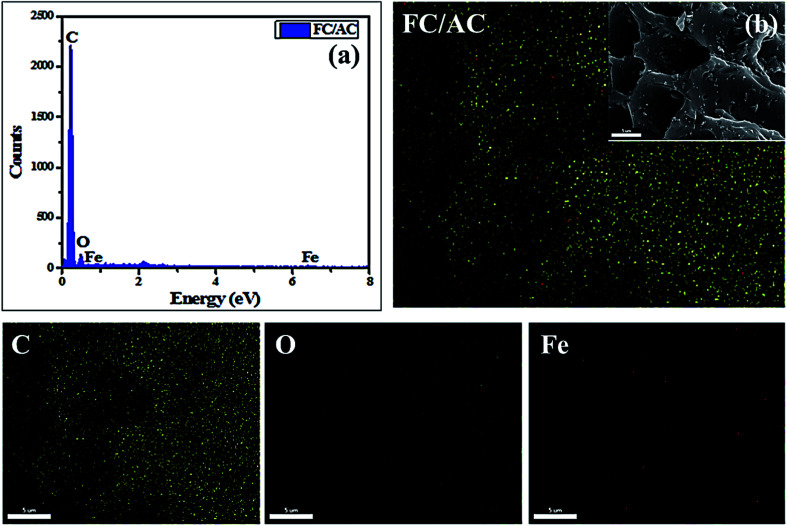
(a) EDAX and (b) mapping analysis of FC/AC.

Further, EDAX analysis (Fig. 5(a)) confirmed the expected elements in the FC/AC catalyst which were present in the stoichiometric amount. Additionally, the ferrocene was uniformly distributed over the AC surface as could be observed from mapping analysis (Fig. 5(b)).

#### 3.1.5. XPS analysis

**Fig. 6:**
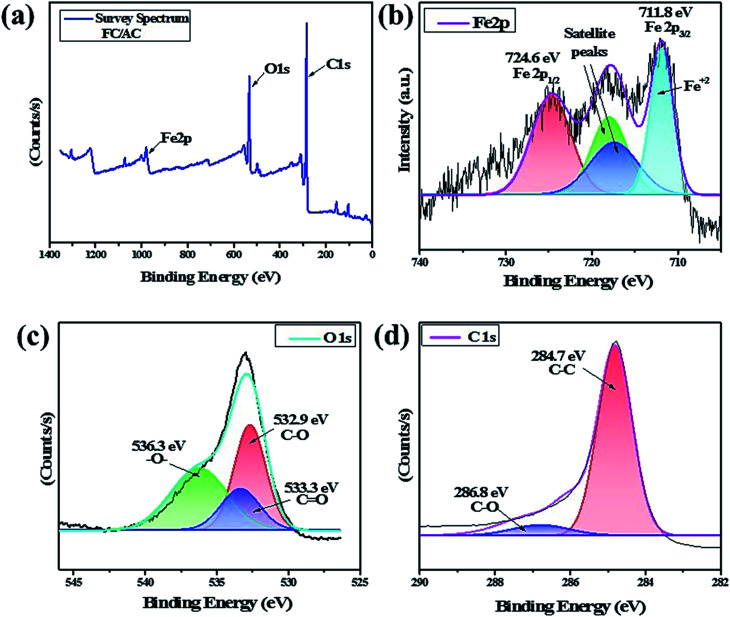
(a) Survey spectrum of FC/AC, deconvulated spectra of (b) Fe2p, (c) O1s and (d) C1s.

The XPS analysis of FC/AC catalyst was performed and the results are displayed in Fig. 6. Fig. 6(a) corresponds to the survey spectrum of FC/AC which confirmed the presence of expected elements such as C, Fe, and O present in the catalyst. Fig. 6(b) displayed the deconvoluted spectrum of Fe2p with a prominent peak at 711.8 eV which could be ascribed to Fe^2+^ arising due to existence of divalent central Fe atom bound to two cyclopentadienyl (C_p_) rings.^45,58,59^ Further, the peaks at 284.5 eV and 532.2 eV observed in the survey spectrum (Fig. 6(a)) could be related to C1s and O1s atoms, respectively, which suggested the presence of activated carbon.

The O1s spectrum was also deconvulated into three peaks as shown in Fig. 6(c). A peak at 536.3 eV correspond to the presence of –O– group suggesting presence of ether, epoxide or hydroxyl groups.^60^ A peak at 533.3 eV was detected due to the presence of C=O groups suggesting presence of carboxylic acid groups. Additionally, occurrence of C–O groups was evident by presence of a peak at 532.9 eV suggesting presence of carbonyl and carboxylic acid functionality over the surface of catalyst. This indicated existence of functional groups over the surface of activated carbon as also supported by FT-IR analysis. Fig. 6(d) showed the core-level spectrum of C1s with a major peak can be detected at 284.3 eV, corresponding to C–C bonds of cyclopentadienyl rings in FC.^61^

#### 3.1.6. BET and BJH analysis

**Fig. 7:**
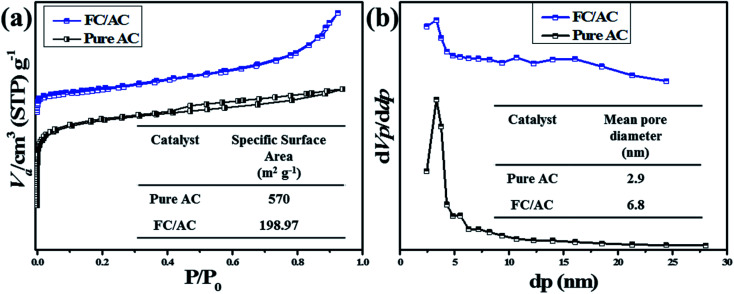
(a) BET plot of pure AC and FC/AC and (b) BJH plot of pure AC and FC/AC.

BET and BJH analysis were performed to investigate the specific surface area and pore size distribution of the materials.^37^ As can be observed from the Fig. 7(a). The BET plot of AC describes a mixed type of isotherm curve according to the IUPAC nomenclature.^62^ Precisely, the isotherm curve at low pressure relates to the type I which is the characteristic of microporous material.^62^ Whereas, at the intermediate and high pressure the obtained curve relates to the type IV isotherm as there is no clear plateau but certain slopes can be observed.^62^ The occurrence of type IV isotherm in AC suggested the transition from microporosity to mesoporosity and therefore displayed high surface area of 540 m^2^ g^−1^.^62^ Further, N_2_-adsorption–desorption curve of FC/AC catalyst displayed type IV isotherm which is a typical feature of mesoporous material.^37^ The BET surface area of FC/AC catalyst decreased with introduction of FC as the pores of AC got filled with FC.

Furthermore, pore size distribution of the materials was evaluated with the assistance of BJH method and the plots are represented in the Fig. 7(b). The mean pore diameter of AC was observed as 2.9 nm. On the other hand, FC/AC catalyst exhibited the mean pore diameter was observed as 6.8 nm. The results obtained from the BET and BJH analysis are represented in the Table 1. The observation has shown good agreement with those reports in the literature.^62^

**Table 1:** Summary of BET surface area, mean pore diameter and total pore volume of pure AC and FC/AC

Entry	Sample	Specific surface area (m^2^ g^−1^)	Mean pore diameter (nm)	Mean pore volume (cm^3^ g^−1^)
1	Pure AC	570	2.9	0.41
2	FC/AC	198.97	6.8	0.33

#### 3.1.7. NH_3_-TPD analysis

**Fig. 8:**
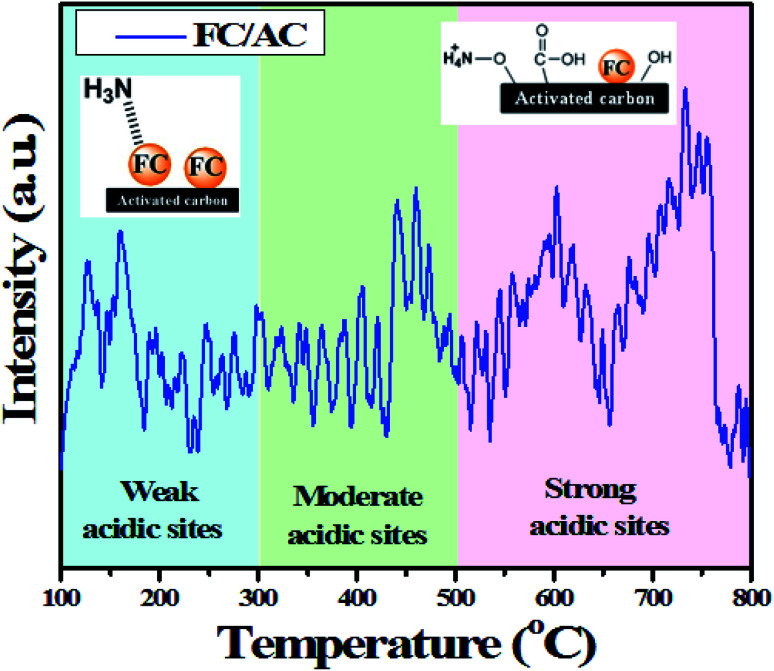
NH_3_-TPD analysis of FC/AC catalyst.

Acidity of the catalyst has an important role to play in bringing about this transformation of ketone and diamine into corresponding 1,5-benzodiazepines.^8,36^ Therefore, NH_3_-TPD analysis was employed to quantify the amount of acidic sites present in the FC/AC catalyst. The NH_3_-TPD profile of FC/AC catalyst is shown in Fig. 8. As can be clearly observed from the profile, prominent peaks were observed in all the three regions. Precisely, the peaks obtained in the temperature range 100–300 °C correspond to the presence of weak acidic sites. Literature suggested that NH_3_ interacting with Lewis acidic sites (metal site) desorbs at low temperature range.

In this regards, NH_3_ interacted with ferrocene metal sites in the FC/AC catalyst suggested presence of weak Lewis acidic sites. The peaks in the temperature range 300–500 °C represented moderate acidic sites.^4^ Whereas, peaks observed above 500 °C were due to presence of strong acidic sites.^4^ As suggested in the literature, NH_3_ interacting with Brønsted acidic sites generates NH_4_^+^ which desorbs at higher temperatures. The amount of acidic sites in the FC/AC catalyst was calculated as 2.2 mmol g^−1^ which could be sufficient in order to bring about effective condensation of diamine and ketone and produce 1,5-benzodiazepine.

### 3.2. Catalytic activity of FC/AC towards synthesis of 1,5-benzodiazepine derivatives

#### 3.2.1. Initial catalytic activity of FC/AC towards synthesis of 1,5-benzodiazepine derivatives

After confirming the successful formation of FC/AC catalysts with the assistance of several analytical and spectroscopic characterization techniques, the prepared catalysts were tested for their catalytic activity towards synthesis of 1,5-benzodiazepine reaction. In this regards, several reactions were initially performed to optimize the catalyst loading amount, effective reaction temperature and effect of solvent to achieve highest conversion, maximum yield and selectivity towards 1,5-benzodiazepine derivatives. To being with, we performed a neat catalyst free condensation reaction between amine (1 eq.) and ketone (2.2 eq.) at 90 °C temperature for 8 h under solvent-free condition in the absence of catalyst to understand the importance of prepared catalyst for selective benzodiazepine synthesis. The neat reaction did not proceed successfully with expected conversion and yield (Table 2, entry 1). Hence, to see the catalytic activity property of FC/AC, the catalysts were prepared with different ferrocene loading and then tested in the for the synthesis of 1,5-benzodiazepine.

**Table 2:** Optimization of reaction conditions with the FC/AC catalyzed 1,5-benzodiazepine synthesis^a^

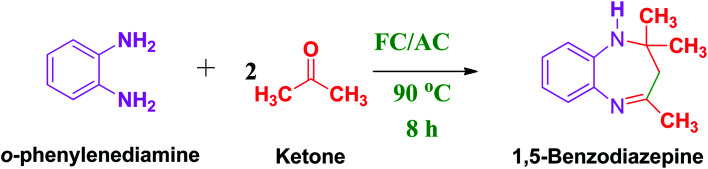				
Entry	Catalyst	Catalyst dosage (wt%)	Conv./sel.^b^ (%)	Yield^b^ (%)
1	Without catalyst	0	0/0	0
2	5 wt% FC/AC	10	57/87	50
3	10 wt% FC/AC	10	99/91	90
4	15 wt% FC/AC	10	98/91	89
5	20 wt% FC/AC	10	98/92	90
6	10 wt% FC/AC	20	90/94	85
7	10 wt% FC/AC	30	94/93	87

a Reaction conditions: all reactions proceeded with 1 mmol *o*-phenylenediamine (OPDA), 2.2 mmol ketone, and FC/AC catalyst under solvent-free condition for 8 h.

b Yield refers to isolated product which characterized by ^1^H NMR, ^13^C NMR.

c Relative polarity of the solvents.

Precisely, ferrocene loading in the FC/AC catalyst was varied from 5 to 20 wt% with respect to the activated carbon. With minimum 5 wt% ferrocene grafted on AC which is denoted as 5 wt% FC/AC catalyst was tested and it resulted in 50% yield towards 1,5-benzodiazepine and 57% conversion of diamine (Table 2, entry 2). The obtained results suggested that 5 wt% FC/AC active towards 1,5-benzodiazepine synthesis but require tuning for the highest conversion and selectivity. Further, increased amount of ferrocene on AC that is 10 wt%, FC/AC catalyst in the same reaction conditions, the reaction resulted remarkable yield of 90%, 91% selectivity towards 1,5-benzodiazepine and 99% conversion of diamine (Table 2, entry 3).

Likewise, on increasing the ferrocene loading to 15 and then 20 wt% on AC and used it for activity study, the reactions revealed constant conversion of diamine and selectivity towards the desired product (Table 2, entries 4, 5). Hence, the obtained results suggested that adequate acidity of the catalyst is essential to proceed the condensation reaction between amine and ketone to obtained efficient conversion, yield and selectivity of the desired product.^8,63^

Meanwhile we have also conducted few reactions with increasing amount of 10 wt% of FC/AC catalyst. These reactions also revealed similar kind of results with constant conversion and reduction in the selectivity of desired product with using high concentration 10 wt% of FC/AC catalyst (Table 2, entries 6, 7). Hence, it is worth to mention here that, 10 wt% of ferrocene loading in FC/AC catalyst provided sufficient active and acidic sites for the reaction to progress in positive direction with appreciable conversion, yield, and selectivity. Therefore, 10 wt% of FC/AC catalyst was considered as an optimum ferrocene loading and catalyst weight for further reactions in this work.

#### 3.2.2. Effect of temperature and effect of different solvents on FC/AC catalyzed synthesis of 1,5-benzodiazepine

Effect of temperature on the catalytic activity of 10 wt% of FC/AC on synthesis of 1,5-benzodiazepine by condensation reaction of aromatic diamine and ketone was studied by varying temperature range from room temperature to 90 °C keeping rest of the reaction parameters constant and the results are shown in Table 3. While considering the green chemistry factor, initially we tried the reaction at room temperature (35 °C), the reaction did not proceed to obtain the desired product and effective conversion (Table 3, entry 1). Hence it is suggested that reaction required temperature to trigger the catalytic path for the required product formation with 10 wt% of FC/AC catalyst. Further, on increasing the reaction temperature to 60 °C, reaction revealed 26% conversion of diamine but did not produce 1,5-benzodiazepine product. It is considered that, diamine is getting interacted with the catalyst surface but failed to produce desired product hence slight conversion observed at 60 °C with catalyst (Table 3, entry 2).

**Table 3:** Optimization of temperature and solvents for the FC/AC catalyzed synthesis of 1,5-benzodiazepine derivatives^a^

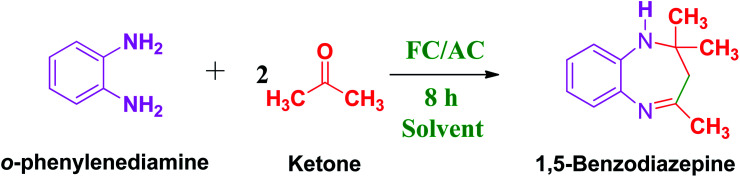				
Entry	Temp (°C)	Solvent (10 mL)	Conv./sel.^b^ (%)	Yield^b^ (%)
1	R.T.	—	0/0	0
2	60	—	26/0	0
3	70	—	42/94	39
4	80	—	62/91	56
5	90	—	99/91	90
6	90	Acetonitrile (0.46)^c^	15/88	13
7	90	Methanol (0.762)^c^	12/88	11
8	90	THF (0.207)^c^	28/90	25
9	90	Chloroform (0.259)^c^	94/93	87
10	90	*n*-Hexane (0.009)^c^	88/95	84

a Reaction conditions: all reactions proceeded with 1 mmol *o*-phenylenediamine (OPDA), 2.2 mmol ketone, and 10 wt% FC/AC catalyst (amount of catalyst-10 wt%) for 8 h.

b Yield refers to isolated product which characterized by ^1^H NMR, ^13^C NMR.

With increasing the reaction temperature at 70 °C and 80 °C, conversion and selectivity was also increased with the 10 wt% of FC/AC catalyst. These reactions showed 42% and 62% conversion, respectively and obtained 94% and 91% selectivity of 1,5-benzodiazepine product but failed to produce efficient yield 39% and 56%, correspondingly (Table 3, entries 3, 4). In the next step, when the reaction was performed at 90 °C, results revealed that, 99% conversion of diamine and 91% and 90% selectivity and yield of 1,5-benzodiazepine product (Table 3, entry 5). Hence it is concluded that, 90 °C reaction temperature is best optimized temperature for the particular reaction with FC/AC catalyst under solvent free condition and it is considered for further study.

Nature of solvent also has a significant influence on the catalytic organic transformation depending on the type of catalyst employed.^63^ Therefore, it is important to investigate the effect of different solvents on the catalytic synthesis of 1,5-benzodiazepine. In addition to the effect of reaction temperature, we have also studied the effect of different solvents on the conversion and selective of 1,5-benzodiazepine product formation. The values for relative polarity are normalized from measurements of solvent shifts of absorption spectra and were extracted from the literature.^64^ In the beginning, acetonitrile was used as solvent with same reaction condition and just 15% conversion and 90% selectivity with 11% yield in 8 h reaction time was obtained (Table 3, entry 6). Meanwhile, 12% conversion, 88% selectivity and 11% yield was obtained in methanol in the uniform reaction condition (Table 3, entry 7). These results suggested that, polar solvents are not much favorable for this reaction and required high amount of time and similar results were obtained by other research groups.

Furthermore, THF and chloroform was used as solvent and obtained 28% and 94% conversion with 90% and 93% selectivity with 25% and 87% yield (Table 3, entries 8, 9). In these reactions, chloroform showed good and effective results towards this reaction nevertheless due to the green chemistry aspects chloroform cannot be a suitable candidate for sustainable process, hence chloroform did not considered for further other aspects in this reaction to obtained 1,5-benzodiazepine product. On the other side, nonpolar solvent *n*-hexane showed 88% conversion and 95% selectivity with 84% yield (Table 3, entry 10). This reaction result also proved that, non-polar solvents were also not up to the mark for efficient conversion, selectivity and yield as compared to the solvent free condition.

As solvent-free synthesis offer several advantages such as less pollution, less handling cost, simpler experimental as well as work-up process, it could be considered as one of the significant step towards green chemistry approach. Overall, several experiments that were performed demonstrated that acidic FC/AC catalyst effectively afforded 1,5-benzodiazepine in high yields under mild and solvent-free conditions making this reaction industrially feasible. Hence, the solvent free condition can be considered as remarkable condition and hence considered as an optimized condition for further applications in this manuscript.

### 3.3. Comparison of present catalytic system with known catalysts for 1,5-benzodiazepine synthesis

To compare the efficiency of the FC/AC catalyst towards synthesis of 1,5-benzodiazepine derivatives with other known homogeneous and heterogeneous catalysts, we performed the same reaction under optimized condition in the presence of different available Fe-based catalysts. The results are summarized in the Table 4. Initially, we began with the Fe powder as a catalyst to yield benzodiazepine under this study. However, Fe powder catalyst displayed very poor activity towards the synthesis of 1,5-benzodiazepine derivatives with only 44% yield, 56% conversion and 78% selectivity (Table 4, entry 1). Additionally, it was very difficult to separate the sticky Fe powder catalyst from the reaction mixture after completion of the reaction. However, the results based on Fe powder catalyzed reaction did not show any further improvement compared with the FC/AC catalyst.

**Table 4:** Synthesis of 1,5-benzodiazepine derivative using different heterogeneous catalyst and their comparison with FC/AC catalyst under optimized reaction condition^a^

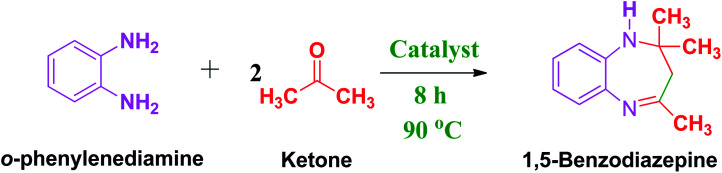			
Entry	Catalyst	Conv./sel.^b^ (%)	Yield^b^ (%)
1	Fe powder	56/78	44
2	FeCl_3_	32/75	24
3	FeCl_2_	38/74	28
4	Fe(NO_3_)_2_	22/74	16
5	Ferrocene	69/92	63
6	Pure activated carbon	47/85	40
7	10 wt% FC/AC	99/91	90

a Reaction conditions: all reactions proceeded with 1 mmol *o*-phenylenediamine (OPDA), 2.2 mmol ketone, and amount of catalyst-10 wt%, at 90 °C under solvent-free condition for 8 h.

b Yield refers to isolated product which characterized by ^1^H NMR, ^13^C NMR.

Next, FeCl_3_ was also employed as a catalyst for the synthesis of 1,5-benzodiazepine derivatives. The reaction resulted in low yield 24% towards the desired product and 32% conversion of diamine (Table 4, entry 2). Moreover, the reaction was complex due to the hygroscopic nature of FeCl_3_ and beside that, FeCl_3_ being homogeneous in nature and it was tedious to separate it out it from the reaction mixture.^65^ We also performed reaction in the presence of FeCl_2_ which displayed similar poor activity with reaction drawbacks similar with FeCl_3_ catalyzed reaction (Table 4, entry 3). Additionally, nitrate precursor of iron (Fe(NO_3_)_2_ – ferric nitrate) was also employed as a catalyst which demonstrated 22% conversion with 16% yield towards 1,5-benzodiazepine (Table 4, entry 4).

Further, in the presence of unsupported ferrocene the reaction progressed with moderate yield (58%) towards the desired product and showed 69% conversion (Table 4, entry 5). We observed improved performance as compared to that of other Fe precursors with catalytic application of ferrocene in the present reaction. However, ferrocene also represented a homogeneous catalytic system which was difficult to separate and recycle from the reaction mixture. On the other side, investigating the activity of pure activated carbon we observed moderate yield (40%) with 47% conversion and 85% selectivity towards the desired product possibly due to acidic nature and functional groups present on activated carbon (Table 4, entry 6).

On the contrary, FC/AC catalyst seemed to be significantly very active and stable catalyst towards the synthesis of 1,5-benzodiazepine derivatives under mild reaction and solvent-free conditions (Table 4, entry 7). The discussion suggested that amongst several iron-based catalysts, supported FC/AC resulted in better catalytic activity possibly due to synergetic effect of highly dispersed ferrocene over the high surface area activated carbon in combination of presence of acidic sites evidenced by characterization techniques.^66^

### 3.4. Synthesis of different 1,5-benzodiazepine derivatives from substituted OPDA and ketones by using FC/AC catalyst

A number of representative substrates was selected in order to explore the influence of electron withdrawing and electron donating groups in the FC/AC mediated condensation of amine and ketone. Under the optimum conditions, we studied the substrate scope with a various set of un-substituted and substituted amines and aromatic, cyclic and acyclic ketones. It was found that all the substrates were viable in this transformation, providing benzodiazepines derivatives in moderate to good yields, which has pharmacological and biological interest. The results has been presented in Table 5.

**Table 5:** Synthesis of different 1,5-benzodiazepine derivatives using FC/AC catalyst under optimized reaction condition^a^

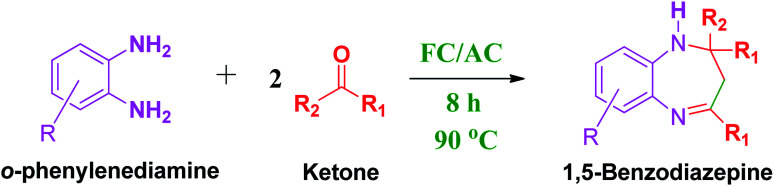						
Entry	Reactant 1 (OPDA)	Reactant 2 (ketone)	Product	Conv./sel. (%)	Yield^b^ (%)	Ref.
1	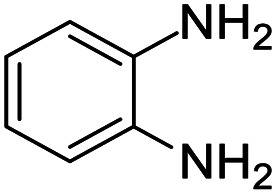	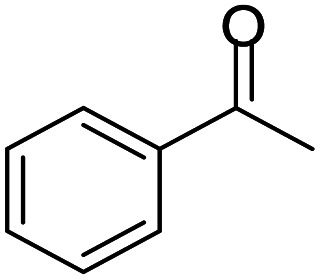	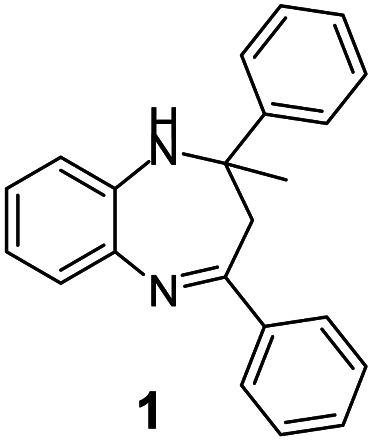	95/85	81	3
2	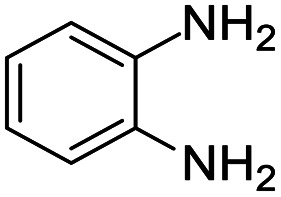	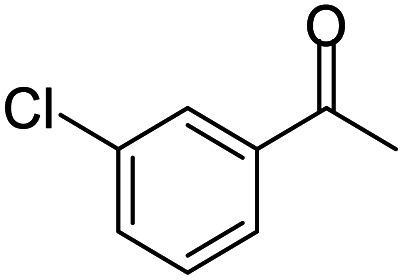	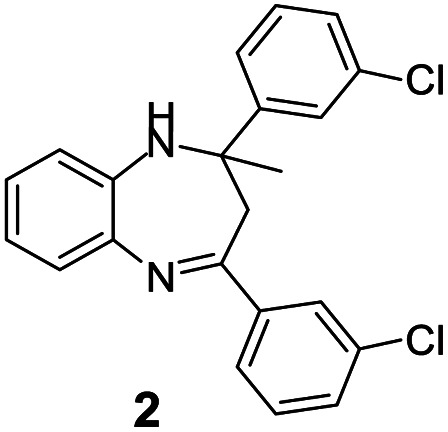	82/88	72	35
3	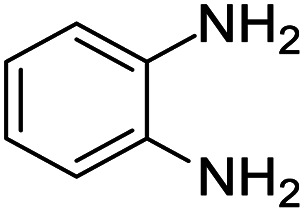	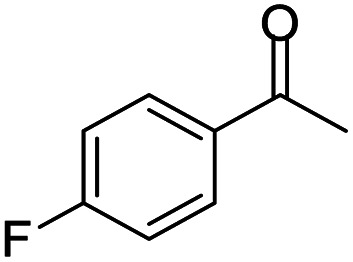	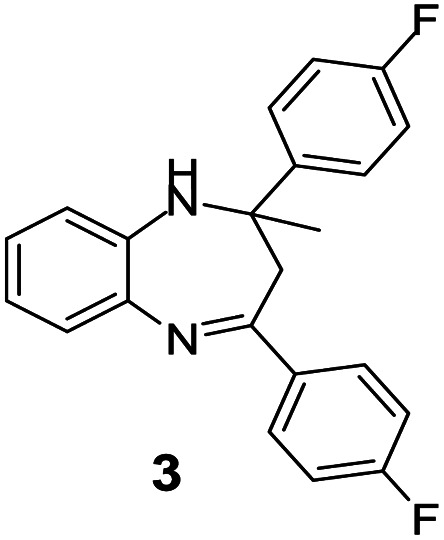	97/91	88	35
4	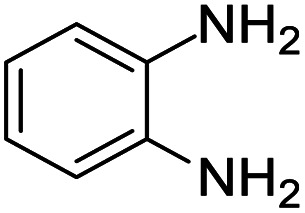	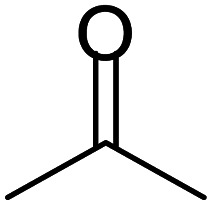	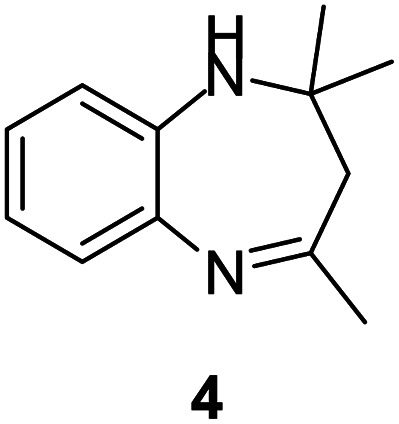	99/91	90	3
5	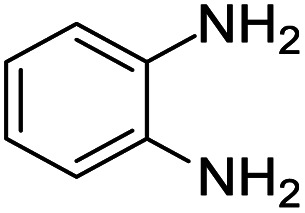	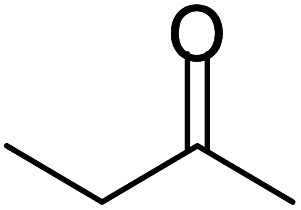	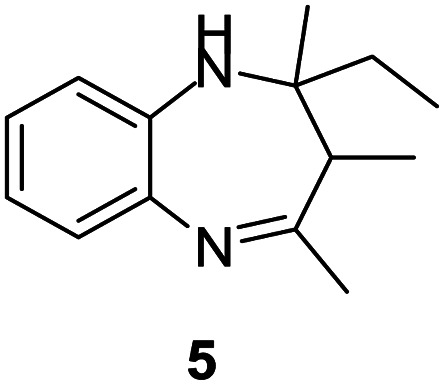	99/89	88	3
6	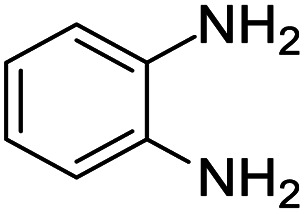	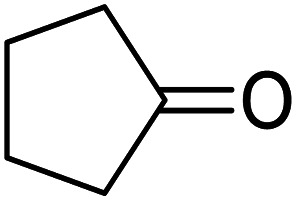	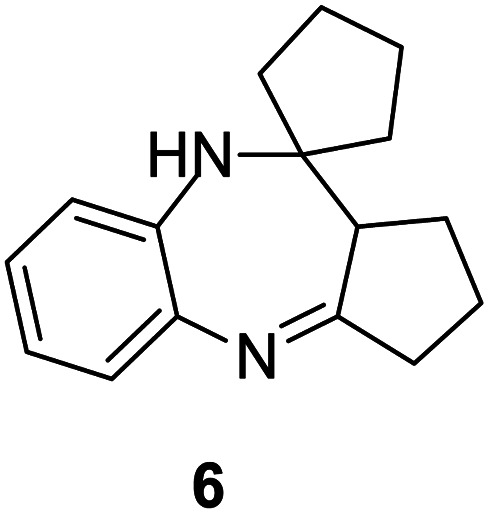	73/89	65	3
7	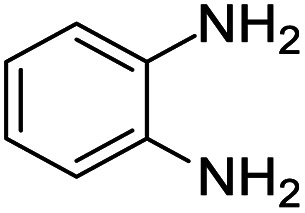	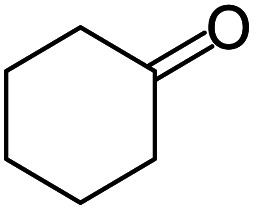	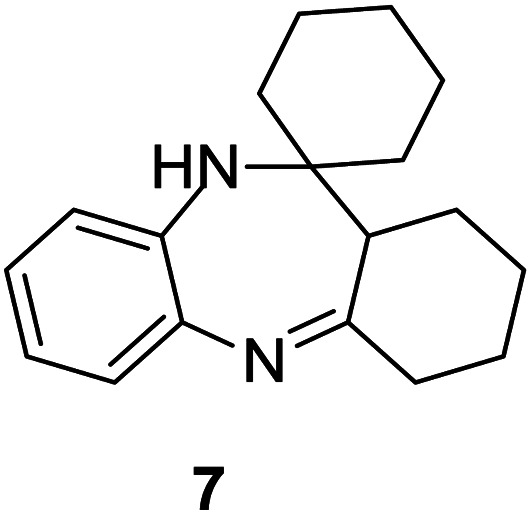	66/92	61	4
8	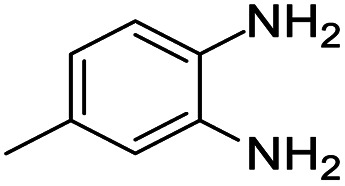	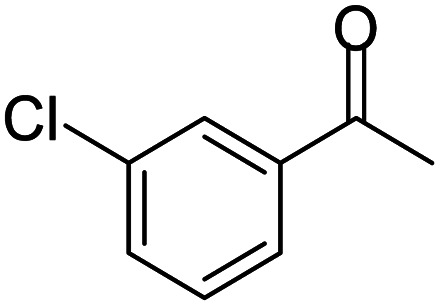	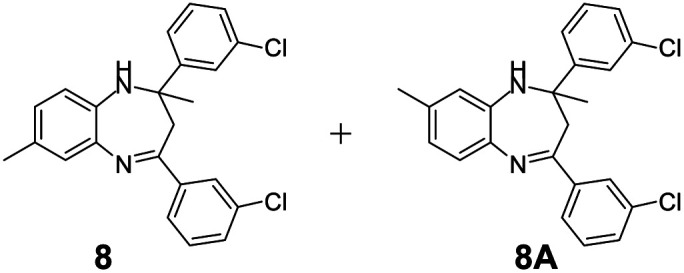	100	87	4
9	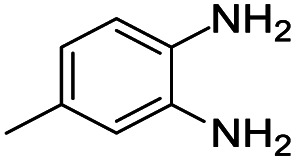	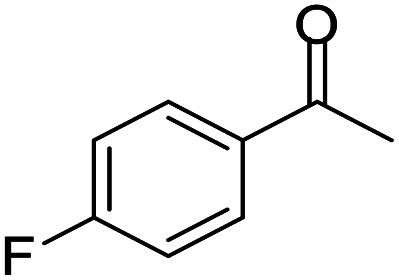	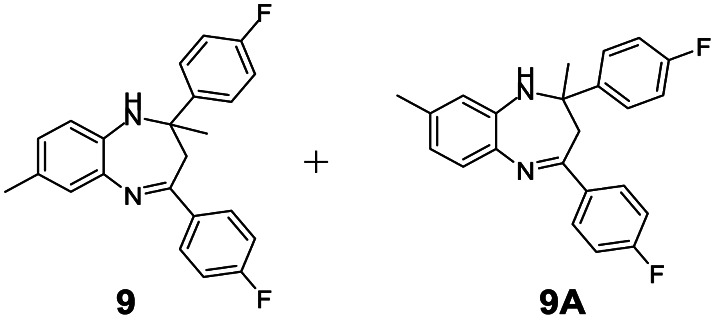	98/92	90	4
10	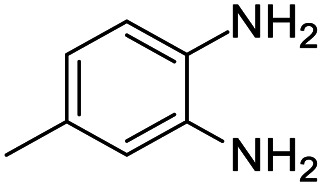	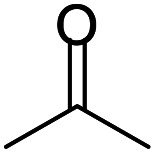	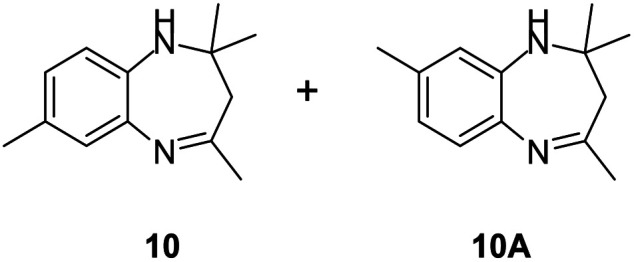	99/85	84	36
11	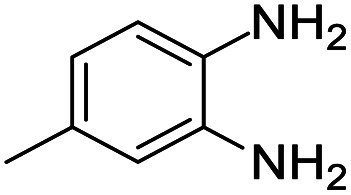	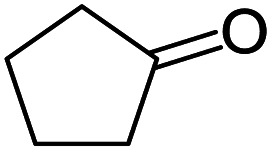	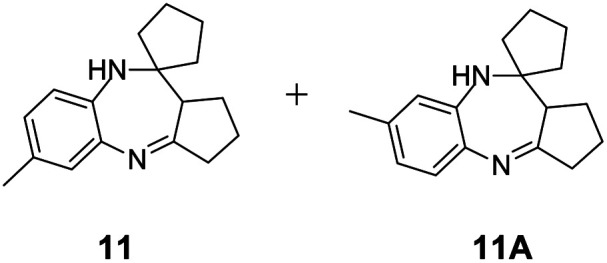	97/88	85	8
12	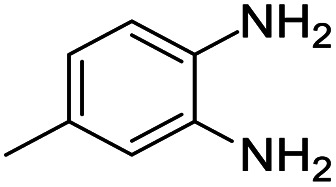	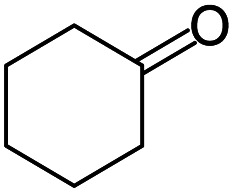	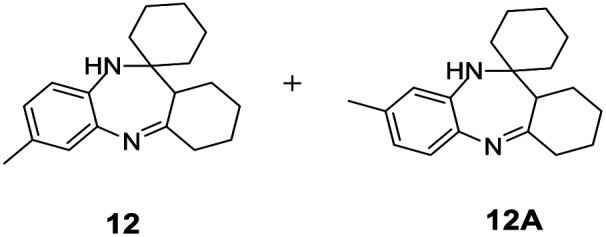	99/91	90	8
13	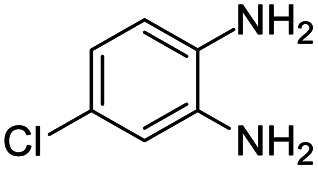	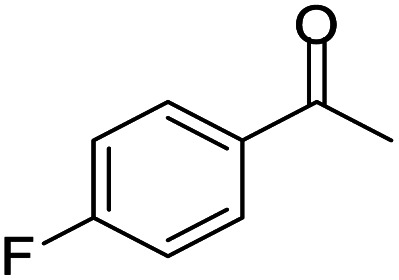	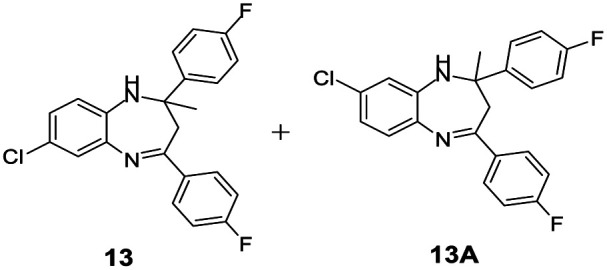	100/90	90	3
14	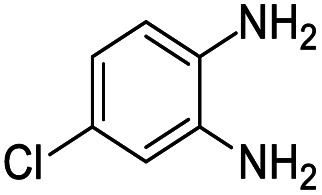	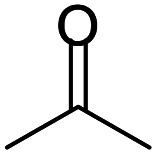	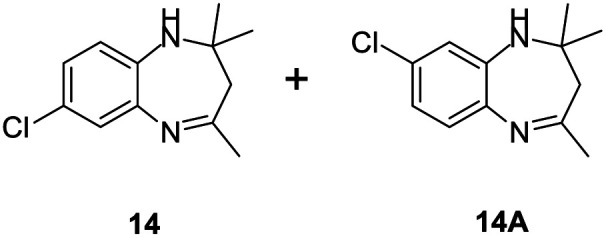	100/87	87	3

a Reaction conditions: all reactions proceeded with 1 mmol substituted *o-*phenylenediamine (OPDA), 2.2 mmol cyclic/acyclic ketone, 10 wt% FC/AC catalyst, 90 °C, 8 h and solvent-free condition.

b Yield refers to isolated product which characterized by ^1^H NMR, ^13^C NMR.

Initially, with un-substituted *o-*phenylenediamine (OPDA) and chloro and fluoro-substituted aromatic ketones under the optimized reaction conditions, the acidic FC/AC resulted in providing the corresponding benzodiazepines derivatives in 72% and 88% yields, respectively (Table 5, entries 2, 3). Further, acyclic ketones and un-substituted OPDA in the presence of FC/AC catalyst presented 90% and 88%, respectively towards the corresponding benzodiazepines derivatives (Table 5, entries 4, 5). On the other hand, with cyclic ketones and un-substituted OPDA we observed moderate yield of 65% and 61%, respectively towards desired benzodiazepine derivatives (Table 5, entries 6, 7).

Further, with electron donating substituted OPDA and electron withdrawing substituted aromatic ketones we observed admirable yields towards corresponding benzodiazepine derivatives (Table 5, entries 8, 9). When methyl substituted OPDA was reacted with aliphatic ketone (acetone) we could afford benzodiazepine derivative with 84% yield (Table 5, entry 10). Interestingly, even with substituted OPDA and cyclic ketone we could achieve 85% and 90% yield towards the corresponding benzodiazepine derivative, respectively (Table 5, entries 11, 12).

The condensation reaction between electron withdrawing substituted OPDA and electron withdrawing substituted aromatic ketone yielded excellent amount of benzodiazepine derivative (Table 5, entry 13). On the other hand, with electron withdrawing substituted OPDA and aliphatic ketone we obtained 87% yield towards the desired product (Table 5, entry 14). The condensation reaction proceeded well in the presence of both electron-withdrawing as well as electron-donating substituents on the di-amine and ketones. Therefore, the reaction showed good functional group tolerance with respect to substituted OPDA and substituted ketones (Table 5, entries 1–14).

### 3.5. Proposed plausible mechanism for FC/AC catalyzed synthesis of 1,5-benzodiazepine

**Scheme 2:**
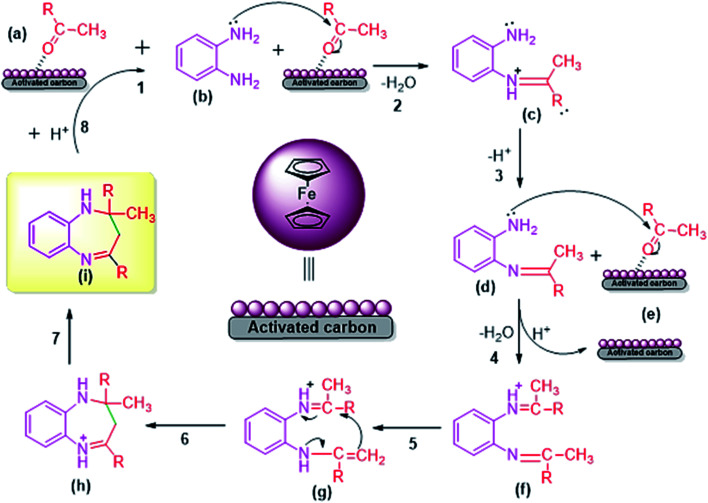
Plausible mechanism for synthesis of 1,5-benzodiazepine derivatives by condensation of OPDA and ketone in the presence of FC/AC catalyst.

The acidic FC/AC catalyst assisted in promoting intramolecular cyclization between OPDA and ketone to form 1,5-benzodiazepine which has been presented in Scheme 2. Initially, the FC/AC catalyst activated ketone (a) which then interacted with *o*-phenylenediamine (b) to form intermediate (c) by elimination of water molecule (step 1–3). Further, the intermediate (d) interact with second molecule of activated ketone (ketone activated over FC/AC catalyst surface) (e) to afford intermediate (f) (step 4). In this step, there was release of catalyst for the new cycle. In the next step, intermediate (f) underwent a 1,3-hydrogen shift at methyl group to form isomeric enamine (g) (step 5). Further, this enamine (g) underwent intramolecular cyclization to afford seven-membered ring (h) and finally the desired product (1,5-benzodiazepine (i)) after which the catalyst is available for next catalytic cycle (step 6–8). The proposed plausible mechanism is well supported by the literature reports.^61–63^

### 3.6. Recyclability studies

**Fig. 9:**
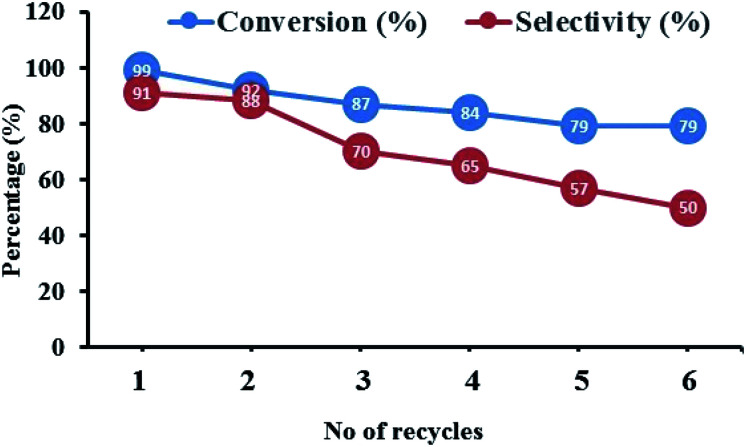
Recyclability performance of FC/AC catalyst for the synthesis of 1,5-benzodiazepines.

The heterogeneous catalysts is of extreme importance in industrial viewpoint as they can be reused for number of catalytic cycles.^67,68^ In order to evaluate the reusability performance of the present catalyst, we performed recyclability study of FC/AC catalyst by merely filtering the solid catalyst from the reaction mixture, giving it a distilled water washing for several times and finally drying under vacuum for 6 h. The dried FC/AC catalyst was then used in the stoichiometric amount and the reaction was performed under optimized reaction conditions. The procedure was repeated until we observed a dramatic decrease in the catalytic performance. The result of the recyclability study is shown in Fig. 9 which demonstrated that the acidic FC/AC catalyst could effectively produce consistent yield towards desired product for up to six consecutive recycles. The decrease in the selectivity after sixth cycle was probably due to formation of uncyclized by-products (stopped at Shiff base stage, step 4 intermediate (f) of mechanism).

Further, the final recycled catalyst was characterized, which revealed, that there were no phase or morphological changes in the catalyst and EDX analysis supported that ferrocene molecules were intact even after consecutive six cycles. The results therefore suggested the catalyst is active and stable and can be efficiently used for the scale-up processes. Therefore the present work exemplifies green approach for synthesis of pharmacological and biologically important 1,5-benzodiazepine derivative under solvent free condition.

## 4. Conclusions

Herein, we reported facile one pot synthesis of acidic ferrocene supported activated carbon (FC/AC) and it was successfully confirmed by using XRD, XPS, FT-IR, FE-SEM, EDX, BET, and NH_3_-TPD analysis. The FC/AC catalyst was investigated for its activity towards the one pot synthesis of 1,5-benzodiazepine derivatives by condensation of *o*-phenylenediamine with ketones (aromatic and aliphatic). The 10 wt% of prepared FC/AC catalyst demonstrated 99% conversion and 91% selectivity towards the desired product under solvent free and mild reaction conditions. In-depth study of effect of reaction parameters such as reaction time, effect of solvents, effect of reaction temperature, effect of different homogeneous and heterogeneous catalysts were also studied. To understand the scope of catalyst in the particular reaction, different substrates were also employed. The catalyst showed appreciable tolerance towards substituted OPDA and cyclic as well as acyclic ketones producing corresponding 1,5-benzodiazepine derivatives in good yields and efficient conversion. Additionally, when unsymmetrical ketones such as 2-butanone was used as one of the substrate, ring closure occurred selectively from one side of the carbon skeleton afforded selective single diazapine product. The reaction products were isolated and identified as 1,5-benzodiazepines. The plausible mechanism for the same has also been elucidated. Moreover, the catalyst displayed appreciable recyclability performance for up to six recycles without significant loss in its catalytic activity. Therefore, the present work highlights a sustainable approach for the synthesis of 1,5-benzodiazepine derivatives in high yields with the assistance of acidic FC/AC as a heterogeneous catalyst under solvent-free condition. The process justified the overarching goals of green chemistry and can be useful for scale-up processes.

## Conflicts of interest

The authors declare that they have no known competing financial interests or personal relationships that could have appeared to influence the work reported in this paper.

## Supplementary Material
